# Clinical Outcomes of HER2-Low Versus HER2-Zero in HR-Positive Metastatic Breast Cancer Treated With Endocrine Therapy With or Without CDK4/6 Inhibitors: A Multicenter Retrospective Study

**DOI:** 10.1155/ijbc/5597051

**Published:** 2025-11-04

**Authors:** Thiti Susiriwatananont, Concord Wongkraisri, Thanate Dajsakdipon, Archara Supavavej, Arunee Dechaphunkul, Patrapim Sunpaweravong, Sunee Neesanun, Suthinee Ithimakin, Thitiya Dejthevaporn, Napa Parinyanitikul, TSCO Breast Oncology Group

**Affiliations:** ^1^Department of Medicine, Faculty of Medicine, King Chulalongkorn Memorial Hospital and Chulalongkorn University, Bangkok, Thailand; ^2^Division of Medical Oncology, Department of Medicine, Faculty of Medicine Siriraj Hospital, Mahidol University, Bangkok, Thailand; ^3^Division of Medical Oncology, Department of Medicine, Faculty of Medicine Ramathibodi Hospital, Mahidol University, Bangkok, Thailand; ^4^Department of Medical Oncology, Chulabhorn Hospital, Chulabhorn Royal Academy, Bangkok, Thailand; ^5^Division of Medical Oncology, Department of Internal Medicine, Faculty of Medicine, Prince of Songkla University, Songkhla, Thailand; ^6^Division of Oncology, Department of Internal Medicine, Sawanpracharak Medical Education Center, in Affiliation With Faculty of Medicine, Praboromrajchanok Institute, Nakhon Sawan, Thailand; ^7^Thai Society of Clinical Oncology, Bangkok, Thailand

**Keywords:** aromatase inhibitors, breast neoplasms, cyclin-dependent Kinase 4/6 inhibitors, receptor, ErbB-2, receptors, estrogen

## Abstract

**Purpose:**

HER2-low status is a predictive factor for novel anti-HER2 therapies in metastatic hormone receptor–positive breast cancer (HR+ MBC). However, its impact on endocrine therapy outcomes remains uncertain. We aimed to explore the effect of HER2-low and HER2-zero status in HR+ MBC patients treated with first-line aromatase inhibitors (AIs) with or without CDK4/6 inhibitors (CDK4/6i).

**Methods:**

We retrospectively reviewed postmenopausal women with HR+ MBC treated with first-line AI ± CDK4/6i between January 1, 2017, and December 31, 2022, from six tertiary hospitals in Thailand. HER2-low was defined as HER2 IHC 1+ or IHC 2+ with ISH-negative. Progression-free survival (PFS) and overall survival (OS) were compared in unadjusted and adjusted cohorts using stabilized inverse probability of treatment weighting (sIPTW), adjusting for age, ECOG performance status, de novo metastasis, endocrine sensitivity, visceral metastasis, number of metastatic sites, and treatment year. Interaction analyses were performed to assess effect modification by HER2 status and other clinical subgroups.

**Results:**

Among 504 patients, 219 (43.5%) were HER2-low, and 285 (56.5%) were HER2-zero. Median follow-up was 31 months (IQR 19–47). CDK4/6i + AI was administered to 52.5% of HER2-low and 43.9% of HER2-zero patients. After sIPTW adjustment, CDK4/6i + AI prolonged median PFS to 22.1 months compared with 21.5 months for AI alone in the HER2-low cohort (HR = 0.80, 95% CI 0.54–1.18; *p* = 0.26) and to 20.1 months compared with 13.5 months in the HER2-zero cohort (HR = 0.65, 95% CI 0.45–0.93; *p* = 0.02). Median OS was 49.3 months with CDK4/6i + AI versus 48.4 months with AI alone in HER2-low (HR = 0.81, 95% CI 0.48–1.36; *p* = 0.43) and 45.8 versus 42.3 months in HER2-zero (HR = 0.85, 95% CI 0.43–1.05; *p* = 0.52). Subgroup analyses showed consistent benefit of CDK4/6i + AI across most clinical categories. The interaction test for treatment × HER2 status was not significant (HR = 1.21, 95% CI 0.74–1.98; *p* = 0.44), indicating no effect modification by HER2-low status.

**Conclusions:**

HER2-low status was not associated with prognosis or predictive value for CDK4/6i efficacy, supporting CDK4/6i + AI as the standard first-line therapy irrespective of HER2 expression level.

## 1. Introduction

Hormone receptor–positive (HR+) breast cancer is the most common subtype, accounting for 50%–70% of all metastatic breast cancer (MBC) diagnosed [1–3]. Cyclin-dependent Kinase 4/6 inhibitors (CDK4/6i) combined with endocrine therapy (ET) are currently recommended as the standard first-line treatment, based on results of landmark Phase 3 studies [4–6]. However, to date, no biomarker can predict the benefits of CDK4/6i therapy other than HR+ status [7, 8].

HER2-low, defined as an immunohistochemistry (IHC) score of 1+ or 2+ with in situ hybridization (ISH) negativity, was reported in up to 71% in HR+ breast cancers and 53% in triple-negative breast cancers (TNBCs) [9, 10]. HER2-low status has emerged as a predictive biomarker for high-potency anti-HER2 antibody–drug conjugates, such as trastuzumab deruxtecan (T-DXd), in MBC, regardless of HR+ status [11]. Nonetheless, the impact of HER2-low status on the prognosis and efficacy of ET and CDK4/6i in HR+ MBC remains uncertain. A previous study in Thai MBC patients reported favorable overall survival (OS) in the HER2-low population compared with HER2-negative patients, irrespective of ET or chemotherapy received [12]. However, other studies reported worse outcomes in patients with HER2-low status [13–16].

We aimed to evaluate the effect of HER2-low and HER2-zero status on treatment outcomes in HR+ MBC patients treated with first-line aromatase inhibitors (AIs) with or without CDK4/6i.

## 2. Materials and Methods

### 2.1. Study Design

This study was a multicenter retrospective cohort study, deriving data from electronic medical records of six tertiary hospitals in Thailand. This study was approved by the Institutional Review Board (IRB) at each participating hospital. Given the retrospective nature of the study and the use of deidentified data, the requirement for informed consent was waived.

### 2.2. Study Patients

Postmenopausal women or premenopausal women who received ovarian function suppression, diagnosed with HR+/HER2− MBC between January 1, 2017, and December 31, 2022, were included. Patients were eligible if they received AI alone or CDK4/6i + AI as first-line therapy. HER2 status was assessed in primary tumor or metastatic tumor specimens by local institutions. Patients were excluded if HER2 status was equivocal or missing. Patients with a follow-up time less than 3 months after the diagnosis of MBC were also excluded, unless they had disease progression or death. Eligible patients were assigned to two cohorts based on their HER2 status. HER2-low was defined as an IHC score of 1+ or 2+ with negative ISH testing, and HER2-zero was defined as an IHC score of 0.

### 2.3. Outcomes

The primary outcome was progression-free survival (PFS), defined as the time from treatment initiation to disease progression or death. Progression was determined based on radiologic criteria (RECIST) and/or clinical assessment. Patients without documented progression were censored at the date of their last follow-up visit or at the data cutoff date. OS was defined as the time from treatment initiation to death from any cause. All deaths were confirmed through the Thai national population registry. Patients who were alive at the data cutoff date were censored.

### 2.4. Statistical Analysis

Categorical variables were presented in frequencies and percentages. Continuous variables were presented as means, standard deviations (SDs), medians, interquartile ranges (IQRs), and ranges. Comparisons between groups were performed in the unadjusted population and after stabilized inverse probability of treatment weighting (sIPTW) to balance patient characteristics. Propensity scores were estimated using a multivariable logistic regression model including age at diagnosis, ECOG performance status, de novo metastasis, endocrine sensitivity, visceral metastasis, number of metastatic sites, and treatment start year (categorized into two calendar periods: 2017–2019 and 2020–2022) to account for calendar-time confounding. Patients who relapsed within 2 years of adjuvant ET were classified as having primary endocrine resistance, whereas those who relapsed while on adjuvant ET after 2 years or within 12 months of completing ET were classified as having secondary endocrine resistance. Covariate balance before and after weighting was assessed using standardized differences (STDs), with values > 0.1 considered indicative of meaningful imbalance. The weighted Kaplan–Meier method was used to estimate median survival times, and weighted Cox proportional hazards models were applied to calculate hazard ratios (HRs) with 95% confidence intervals (CIs). All analyses were conducted using Stata Version 18 (StataCorp, 2023) and R Version 4.1.2 (R Core Team, 2021).

## 3. Results

**Figure 1:**
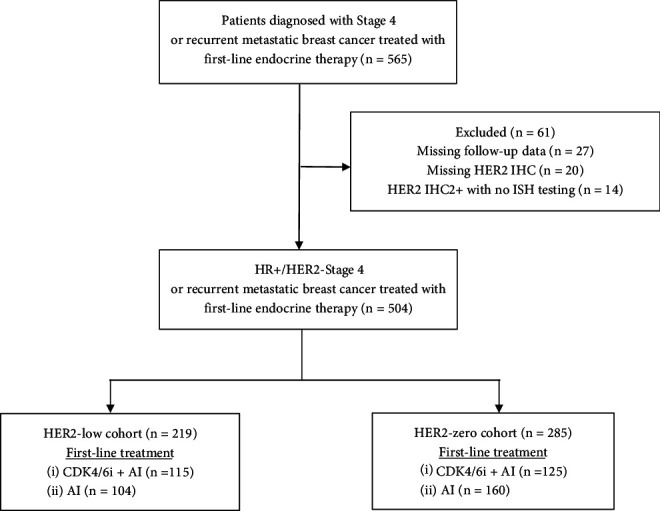
Patient disposition diagram.

A total of 504 patients with HR+/HER2-negative MBC diagnosed between January 1, 2017, and December 31, 2022, were included. Of these, 219 patients (43.5%) were classified as HER2-low and 285 patients (56.5%) as HER2-zero (Figure 1). Overall, 489 patients (97%) had estrogen receptor (ER) expression > 10%. Approximately half of the patients (47.6%) received CDK4/6i + AI as first-line treatment, with ribociclib being the most commonly prescribed CDK4/6i (72.1%).

Before weighting, there were notable imbalances in baseline characteristics between treatment groups, including age, endocrine sensitivity, metastatic sites, and treatment start year (Tables 1 and 2). After sIPTW adjustment, covariate balance was achieved across most characteristics, with standardized mean differences approaching zero (Supporting Information 1: Figure S1). The sIPTW weights had a mean of 1.00 (SD 0.67), ranging from 0.53 to 6.34, indicating good overlap between treatment groups and absence of extreme weights.

After weighting, the mean age at advanced breast cancer diagnosis was 60 years in both cohorts. Forty-five percent of patients presented with de novo metastatic disease, and approximately one-fourth of patients with recurrent disease had endocrine-sensitive disease. At the data cutoff date of September 30, 2023, the weighted median follow-up duration was 30–33 months for patients treated with CDK4/6i + AI and 31–32 months for those receiving AI alone.

### 3.1. PFS

**Figure 2:**
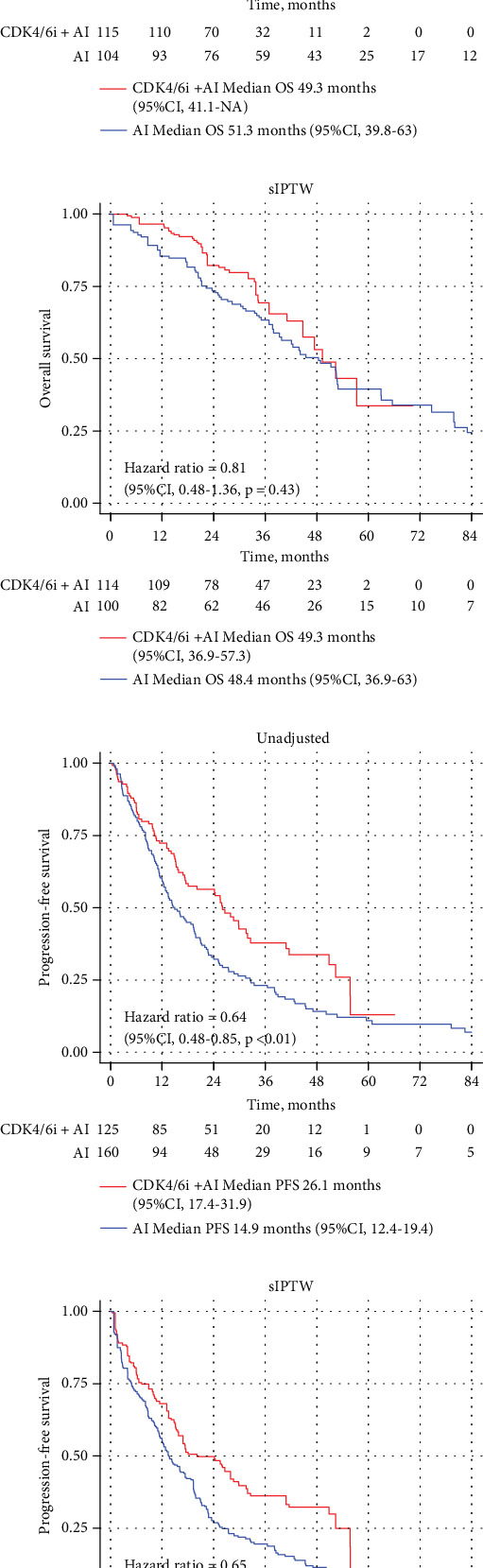
Progression-free survival and overall survival in (a–d) HER2-low and (e–h) HER2-zero in unadjusted cohorts and after sIPTW.

In the unadjusted analysis, median PFS with CDK4/6i + AI was significantly longer compared to AI alone in the HER2-zero cohort (26.1 vs. 16.9 months, HR = 0.64 [95% CI 0.48–0.85], *p* < 0.01), whereas no significant difference was observed in the HER2-low cohort (25.2 vs. 22.3 months, HR = 0.91 [95% CI 0.65–1.27], *p* = 0.56) (Figure 2a,e). After sIPTW adjustment, CDK4/6i + AI remained associated with significantly prolonged PFS in the HER2-zero cohort (20.1 vs. 13.5 months, HR = 0.65 [95% CI 0.45–0.93], *p* = 0.02). In the HER2-low cohort, median PFS was numerically longer with CDK4/6i + AI (22.1 vs. 21.5 months), but the difference was not statistically significant (HR = 0.80 [95% CI 0.54–1.18], *p* = 0.26) (Figure 2b,f).

### 3.2. OS

In the unadjusted analysis, there was no significant difference in median OS between CDK4/6i + AI and AI alone in either the HER2-low cohort (49.3 vs. 51.3 months, HR = 0.88, 95% CI 0.56–1.38, *p* = 0.58) or the HER2-zero cohort (55.7 vs. 42.3 months, HR = 0.76, 95% CI 0.53–1.08, *p* = 0.12) (Figure 2c,g). After sIPTW adjustment, median OS remained numerically longer with CDK4/6i + AI in the HER2-low cohort (49.3 vs. 48.4 months, HR = 0.81, 95% CI 0.48–1.36, *p* = 0.43) and in the HER2-zero cohort (45.8 vs. 42.3 months, HR = 0.85, 95% CI 0.43–1.05, *p* = 0.52), although neither reached statistical significance (Figure 2d,h).

For patients treated with first-line CDK4/6i + AI or AI alone, there were no significant differences in both PFS and OS between HER2-low and HER2-zero cohorts after sIPTW adjustment (Supporting Information 2: Figure S2 and Supporting Information 3: Figure S3).

### 3.3. Interaction and Subgroup Analyses

**Figure 3:**
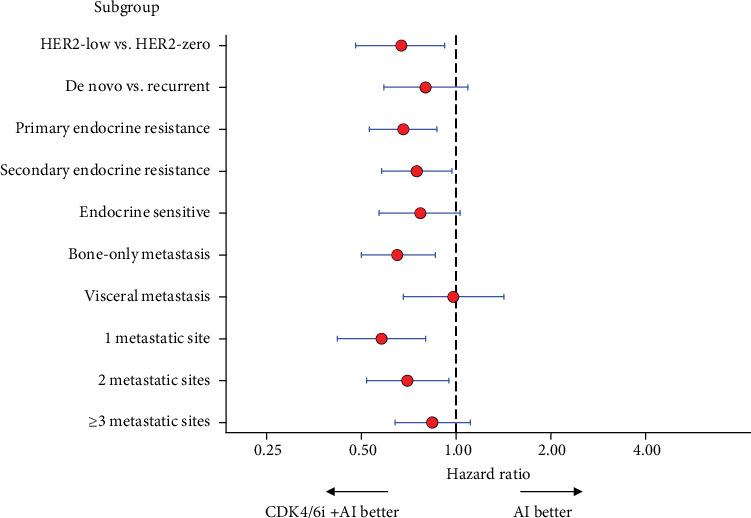
Forest plot of subgroup analyses of progression-free survival with CDK4/6i + AI versus AI after sIPTW adjustment.

Subgroup analyses were performed to explore whether treatment effects differed across clinically relevant populations (Figure 3 and Table 3). After sIPTW adjustment, CDK4/6i + AI was consistently associated with a reduced risk of progression or death across most subgroups. In the HER2-low versus HER2-zero analysis, treatment with CDK4/6i + AI significantly improved PFS (HR 0.67, 95% CI 0.48–0.92; *p* = 0.015). However, the formal interaction test between treatment and HER2 status was not statistically significant (interaction HR 1.21, 95% CI 0.74–1.98; *p* = 0.44), indicating that the apparent difference between HER2-low and HER2-zero cohorts is unlikely to reflect a true modification of treatment effect. Other subgroup analyses, including disease presentation (de novo vs. recurrent), endocrine sensitivity, visceral involvement, and number of metastatic sites, similarly showed no significant interaction.

### 3.4. Subsequent Treatment

Most patients (90.2%) who had disease progression received subsequent treatment (Table 4). Endocrine monotherapy (46.9%) and chemotherapy (34%) were the two most common second-line treatments. Only 8.3% of patients who received first-line AI were treated with CDK4/6i + AI as second-line treatment.

## 4. Discussion

In this multicenter retrospective study of 504 patients with HR+/HER2− MBC, the addition of CDK4/6i to AI was associated with improved outcomes compared to AI alone after adjustment with sIPTW. HER2-low status was not identified as a prognostic factor or a predictive biomarker for CDK4/6i efficacy.

While the superiority of CDK4/6i + AI over AI monotherapy has been consistently established in Phase 3 clinical trials, our study contributes additional evidence from a real-world Southeast Asian population and specifically addresses the role of HER2-low disease. In unadjusted analyses, CDK4/6i + AI significantly prolonged PFS in the HER2-zero cohort but not in the HER2-low cohort. After sIPTW adjustment for baseline imbalances, CDK4/6i + AI improved PFS in both subgroups, with statistical significance observed only in HER2-zero. Importantly, the formal interaction test for treatment × HER2 status was not significant (interaction HR 1.21, 95% CI 0.74–1.98; *p* = 0.44), indicating that the apparent difference in subgroup HRs is unlikely to represent a true biological distinction. Instead, this pattern likely reflects chance variation, residual confounding, and limited statistical power due to the smaller HER2-low sample size. In addition, patients with HER2-low disease who received AI alone tended to have longer follow-up and were more often treated in earlier years (2017–2019), whereas most patients receiving CDK4/6i were treated later (2020–2022). Although weighting corrected most of these imbalances, shorter follow-up in the CDK4/6i arm may have diluted the observed benefit in the HER2-low subgroup.

Our findings align with several prior reports showing no clear prognostic or predictive role of HER2-low in HR+ MBC [17–20] but differ from studies suggesting HER2-low disease carries inferior outcomes [13–16].

The largest meta-analysis, which included 2705 HR+ MBC patients receiving CDK4/6i + AI, revealed a higher risk of progression with HER2-low status (HR 1.22, 95% CI 1.10–1.35, *p* < 0.001). However, most studies included were retrospective with heterogeneous treatment lines and lacked adjustment for key baseline variables [16]. Discrepancies across studies likely reflect heterogeneity in ER expression, endocrine sensitivity, treatment-line composition, and follow-up duration. In our cohort, most patients had high ER expression (> 10%) and relatively few endocrine resistance patients, which may have attenuated the differences between HER2-low and HER2-zero tumors.

In current practice, ER expression level and prior endocrine sensitivity remain more relevant predictors of benefit from ET and CDK4/6 inhibition than HER2 status. ER-low expression (1%–9%) is a biologically distinct group associated with poorer response and may overlap with the basal-like intrinsic subtype [21, 22]. An analysis of intrinsic subtypes from the Phase 3 MONALEESA studies reported that HR+ breast cancers classified as HER2-enriched subtype were associated with improved outcomes, while the basal-like subtype had worse outcomes [23]. These findings underscore the complexity of CDK4/6 biology and the limitations of simple biomarker assessments, highlighting the need for novel predictive biomarkers [8].

Following CDK4/6i + AI therapy, biomarker-driven therapies targeting actionable mutations have demonstrated improved outcomes. Alpelisib and capivasertib in combination with fulvestrant improved PFS in patients with PIK3CA mutation (and AKT1/PTEN alterations for capivasertib) when compared to fulvestrant alone [24, 25]. Elacestrant has shown superiority over fulvestrant or AI in patients with ESR1 mutations [26]. Furthermore, for endocrine-resistant patients with germline BRCA mutations, PARP inhibitors such as olaparib and talazoparib were superior to physician's choice of chemotherapy [27, 28]. HER2-low status currently serves as a predictive factor for T-DXd efficacy in endocrine refractory in HR+ MBC and TNBC following prior chemotherapy [11]. Recently, the DESTINY-Breast06 study further demonstrated the superiority of T-DXd over chemotherapy even in HER2 ultralow disease [29]. As high-potency anti-HER2 therapies become more widely accessible, outcomes for patients with HER2-low breast cancer are expected to improve significantly.

Strengths of this study include the relatively large sample size, inclusion of patients from multiple academic centers, and the use of sIPTW to reduce confounding. The median follow-up of more than 30 months strengthens survival estimates.

### 4.1. Limitations

This study has several limitations. First, the retrospective design cannot eliminate residual confounding. Second, HER2 testing was performed locally on either primary or metastatic tissue without central pathology review, raising the possibility of interinstitutional variability, misclassification, and discordance between primary and metastatic samples. Third, only a small proportion of patients who began treatment with AI alone subsequently received CDK4/6i, which may have biased OS results by underestimating the benefit of CDK4/6i in the AI cohort. Finally, the later introduction of CDK4/6i (post-2020) resulted in shorter follow-up in this group, which may have attenuated observed survival differences.

Despite these limitations, our results confirm the clinical benefit of CDK4/6i + AI in HR+/HER2− MBC and reinforce that HER2-low expression should not influence first-line treatment decisions. Although subgroup HRs appeared more favorable in HER2-zero patients, the nonsignificant interaction analysis demonstrates that the treatment effect of CDK4/6i does not differ by HER2 status.

## 5. Conclusions

HER2-low status was not associated with prognosis or predictive value for CDK4/6i efficacy, supporting the use of CDK4/6i + AI as the standard first-line therapy irrespective of HER2 expression level. Our study adds novel evidence from Southeast Asia, where real-world data remain scarce, and reinforces that treatment decisions for HR+/HER2-negative MBC should not be influenced by HER2-low status.

## Figures and Tables

**Table 1:** Clinical characteristics of the HER2-low cohort before and after sIPTW adjustment.

**Characteristics**	**All patients (** **n** = 219**)**	**Unadjusted**			**After sIPTW**		
		**CDK4/6 + AI (** **n** = 115**)**	**AI (** **n** = 104**)**	**STD**	**CDK4/6 + AI (** **n** = 114**)**	**AI (** **n** = 100**)**	**STD**
Age (median), years	60 (52–68)	59 (48–68)	62.5 (53–68.5)	−0.184	59 (48–68)	60 (50–67)	−0.027
Age (mean, SD), years	60 (12)	58 (12)	61 (12)		59 (12)	59 (12)	
Age group (*n*, %)							
• 18–49 years	47 (21.5%)	29 (25.2%)	18 (17.3%)	0.193	29 (25%)	24 (24%)	0.033
• 50–64 years	93 (42.5%)	51 (44.3%)	42 (40.4%)	0.08	45 (39.5%)	39 (39%)	0.010
• ≥ 65 years	79 (36%)	35 (30.5%)	44 (42.3%)	−0.248	40 (35.4%)	37 (37%)	−0.040
ECOG (*n*, %)							
• 0	67 (30.6%)	35 (30.4%)	32 (30.8%)	−0.007	39 (33.9%)	33 (33%)	0.015
• 1	122 (55.7%)	62 (53.9%)	60 (57.7%)	−0.076	60 (52.3%)	53 (53%)	−0.018
• 2–3	30 (13.7%)	18 (15.7%)	12 (11.5%)	0.12	15 (13.2%)	14 (14%)	−0.006
De novo (*n*, %)	102 (46.6%)	51 (44.3%)	51 (49%)	−0.093	48 (42.1%)	45 (45%)	−0.052
Primary endocrine resistance (*n*, %)	17 (14.5%)	7 (6.1%)	10 (9.6%)	−0.131	11 (9.6%)	9 (9%)	0.023
Secondary endocrine resistance (*n*, %)	31 (26.5%)	19 (16.6%)	12 (11.6%)	0.143	18 (15.8%)	13 (13%)	0.065
Endocrine sensitive (*n*, %)	57 (48.7%)	32 (27.8%)	25 (24%)	0.086	29 (25.4%)	27 (27%)	−0.027
Unknown endocrine sensitivity (*n*, %)	12 (10.3%)	6 (5.2%)	6 (5.8%)	−0.024	8 (7.1%)	6 (5.3%)	0.035
Bone-only metastasis (*n*, %)	53 (24.2%)	24 (20.9%)	29 (27.9%)	−0.163	25 (21.7%)	25 (25%)	−0.078
Visceral metastasis (*n*, %)	123 (56.2%)	65 (56.5%)	58 (55.8%)	0.015	69 (60.7%)	61 (61%)	−0.012
Number of metastatic sites (*n*, %)							
• 1	110 (50.2%)	58 (50.4%)	52 (50%)	0.009	65 (57%)	52 (52%)	−0.086
• 2	64 (29.2%)	34 (29.6%)	30 (28.9%)	0.016	29 (25.4%)	28 (18%)	−0.059
• ≥ 3	39 (17.9%)	21 (18.3%)	18 (17.3%)	0.025	19 (16.7%)	18 (18%)	−0.02
• Unknown	6 (2.7%)	2 (1.7%)	4 (3.8%)	−0.128	1 (0.9%)	2 (2%)	−0.074
Treatment start year (*n*, %)							
• 2017–2019	105 (47.9%)	27 (23.5%)	78 (75%)	−1.197	54 (47.6%)	50 (50%)	−0.052
• 2020–2022	114 (52.1%)	88 (76.5%)	26 (25%)	1.197	60 (52.4%)	50 (50%)	0.052
Median follow-up time (IQR), months	32 (20–47)	29 (19–37)	38.5 (21.5–58)	−0.603	33 (21–46)	32 (18–48)	−0.105

Abbreviations: ECOG, Eastern Cooperative Oncology Group performance status; IQR, interquartile range; SD, standard deviation; sIPTW, stabilized inverse probability of treatment weighting.

**Table 2:** Clinical characteristics of the HER2-zero cohort before and after sIPTW adjustment.

**Characteristics**	**All patients (** **n** = 285**)**	**Unadjusted**			**After sIPTW**		
		**CDK4/6 + AI (** **n** = 125**)**	**AI (** **n** = 160**)**	**STD**	**CDK4/6 + AI (** **n** = 122**)**	**AI (** **n** = 165**)**	**STD**
Age (median), years	61 (54–68)	61 (52–67)	62 (55–69)	−0.242	61 (54–66)	59 (51–67)	0.053
Age (mean, SD), years	60 (12)	59 (12)	62 (11)		59 (12)	58 (12)	
Age group (*n*, %)							
• 18–49 years	47 (16.5%)	26 (20.8%)	21 (13.1%)	0.204	21 (17.2%)	37 (22.3%)	−0.129
• 50–64 years	128 (44.9%)	57 (45.6%)	71 (44.4%)	0.025	62 (51.2%)	72 (43.9%)	0.146
• ≥ 65 years	110 (38.6%)	42 (33.6%)	68 (42.5%)	−0.183	39 (31.7%)	56 (33.8%)	−0.0046
ECOG (*n*, %)							
• 0	64 (22.5%)	39 (31.2%)	25 (15.6%)	0.373	31 (25%)	43 (26.3%)	−0.029
• 1	185 (64.9%)	72 (57.6%)	113 (70.6%)	−0.273	72 (59%)	102 (61.8%)	−0.063
• 2–3	36 (12.6%)	14 (11.2%)	22 (13.8%)	−0.077	19 (16%)	20 (11.9%)	0.126
De novo (*n*, %)	129 (45.3%)	59 (47.2%)	70 (43.8%)	0.069	54 (44.5%)	82 (49.9%)	−0.107
Primary endocrine resistance (*n*, %)	17 (10.9%)	5 (4%)	12 (7.5%)	−0.15	12 (9.7%)	10 (6%)	0.135
Secondary endocrine resistance (*n*, %)	36 (23.1%)	17 (13.6%)	19 (11.9%)	0.051	16 (13.3%)	20 (12.3%)	0.03
Endocrine sensitive (*n*, %)	87 (55.8%)	41 (32.8%)	46 (28.8%)	0.088	37 (30%)	44 (26.6%)	0.075
Unknown endocrine sensitivity (*n*, %)	16 (10.2%)	3 (2.4%)	13 (8.1%)	−0.258	3 (2.6%)	9 (5.2%)	−0.138
Bone-only metastasis (*n*, %)	75 (26.3%)	28 (22.4%)	47 (29.4%)	−0.159	37 (30.5%)	48 (29.1%)	0.031
Visceral metastasis (*n*, %)	168 (59%)	82 (65.6%)	86 (53.8%)	0.243	67 (54.9%)	94 (56.9%)	−0.041
Number of metastatic sites (*n*, %)							
• 1	134 (47%)	51 (40.8%)	83 (51.9%)	−0.223	63 (51.5%)	82 (49.9%)	0.033
• 2	90 (31.6%)	41 (32.8%)	49 (30.6%)	0.047	33 (27.3%)	43 (26.1%)	0.027
• ≥ 3	58 (20.4%)	32 (25.6%)	26 (16.3%)	0.230	25 (20.1%)	38 (23%)	−0.07
• Unknown	3 (1%)	1 (0.8%)	2 (1.3%)	−0.045	1 (0.9%)	2 (1%)	0.004
Treatment start year (*n*, %)							
• 2017–2019	169 (59.3%)	43 (34.4%)	126 (78.8%)	−0.990	71 (58.5%)	95 (57.7%)	−0.016
• 2020–2022	116 (40.7%)	82 (65.6%)	34 (21.2%)	0.990	51 (41.5%)	70 (42.3%)	0.016
Median follow-up time (IQR), months	30 (19–47)	28 (17–42)	33.5 (20–50)	−0.312	30 (19–49)	31 (17–48)	0.214

Abbreviations: ECOG, Eastern Cooperative Oncology Group performance status; IQR, interquartile range; SD, standard deviation; sIPTW, stabilized inverse probability of treatment weighting.

**Table 3:** Subgroup analyses of progression-free survival with CDK4/6i + AI versus AI after sIPTW adjustment.

**Subgroup**	**HR**	**95% CI**	**p** ** value**	**Interaction HR**	**95% CI**	**p** ** value**
HER2-low vs. HER2-zero	0.67	0.48–0.92	0.015	1.21	0.74–1.98	0.437
De novo vs. recurrent	0.80	0.59–1.09	0.153	0.79	0.49–1.29	0.349
Primary endocrine resistance	0.68	0.53–0.87	0.003	2.23	0.99–5.01	0.051
Secondary endocrine resistance	0.75	0.58–0.97	0.027	0.77	0.37–1.56	0.464
Endocrine sensitive	0.77	0.57–1.03	0.082	0.81	0.50–1.32	0.401
Bone-only metastasis	0.65	0.50–0.86	0.002	1.44	0.83–2.50	0.195
Visceral metastasis	0.98	0.68–1.42	0.922	0.58	0.36–0.94	0.028
1 metastatic site	0.58	0.42–0.80	0.001	1.51	0.93–2.44	0.097
2 metastatic sites	0.70	0.52–0.95	0.020	1.10	0.66–1.82	0.713
≥ 3 metastatic sites	0.84	0.64–1.11	0.222	0.40	0.23–0.71	0.002

**Table 4:** Subsequent treatments and chemotherapy used after progression.

**Subsequent treatment**	**HER2-low**		**HER2-zero**	
	**CDK4/6i + AI (** **n** = 68**)**	**AI (** **n** = 81**)**	**CDK4/6i + AI (** **n** = 71**)**	**AI (** **n** = 136**)**
Any second-line treatment	61 (89.7%)	79 (97.5%)	59 (83.1%)	122 (89.7%)
Endocrine monotherapy	30 (44.1%)	48 (59.3%)	30 (42.3%)	59 (43.4%)
CDK4/6 inhibitor + endocrine therapy	1 (1.5%)	9 (11.1%)	—	9 (6.6%)
Endocrine therapy + *PIK3CA* inhibitor	1 (1.5%)	—	2 (2.8%)	—
Chemotherapy	24 (35.3%)	21 (25.9%)	22 (31%)	54 (39.7%)
Chemotherapy in any line of treatment	37 (54.4%)	50 (61.7%)	36 (50.7%)	101 (74.3%)
Missing	5 (7.3%)	1 (1.2%)	5 (7%)	—

## Data Availability

The data that support the findings of this study are available on request from the corresponding author. The data are not publicly available due to privacy or ethical restrictions.

## Supporting Information

Supporting Information 1 Figure S1: The standardized difference values before and after sIPTW in the HER2-low and HER2-zero cohorts.

Supporting Information 2 Figure S2: Differences in Kaplan–Meier curves for progression-free survival and overall survival between the HER2-low and HER2-zero cohorts in patients receiving CDK4/6 + AI and AI alone.

Supporting Information 3 Figure S3: The standardized difference values before and after sIPTW in the CDK4/6i + AI and AI alone cohorts.
