# Supplementary material for: Clinical Outcomes of HER2-Low Versus HER2-Zero in HR-Positive Metastatic Breast Cancer Treated With Endocrine Therapy With or Without CDK4/6 Inhibitors: A Multicenter Retrospective Study
Source: Int J Breast Cancer. 2025 Nov 4;2025:5597051. doi: 10.1155/ijbc/5597051 (PMC12605866; doi:10.1155/ijbc/5597051)
Supplement: Supporting Information 1 — Figure S1: The standardized difference values before and after sIPTW in the HER2-low and HER2-zero cohorts. [file 5597051.f1.pptx]

## Slide 1
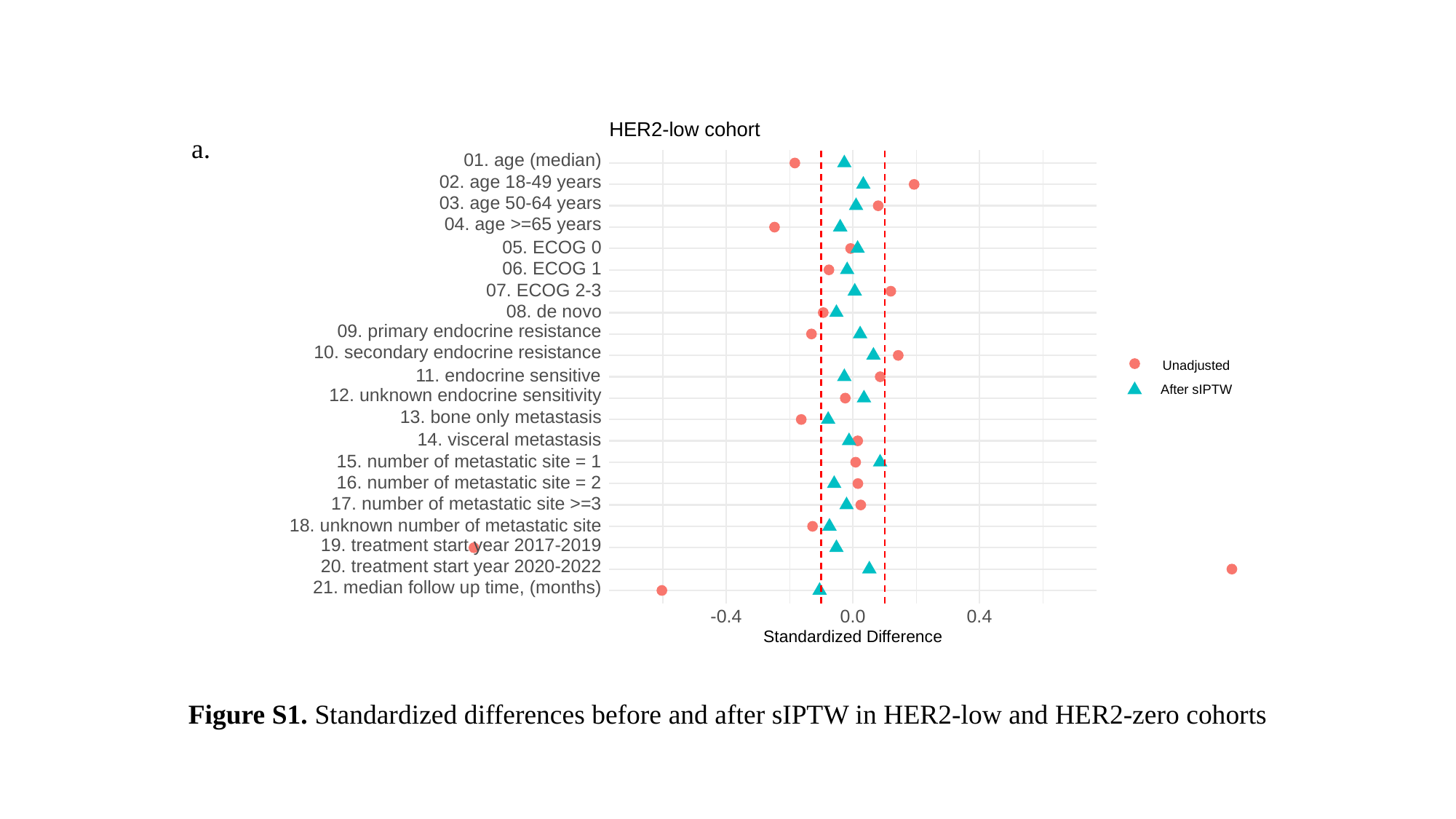

HER2-low cohort
01. age (median)
02. age 18-49 years
03. age 50-64 years
04. age >=65 years
05. ECOG 0
06. ECOG 1
07. ECOG 2-3
08. de novo
09. primary endocrine resistance
10. secondary endocrine resistance
11. endocrine sensitive
12. unknown endocrine sensitivity
13. bone only metastasis
14. visceral metastasis
15. number of metastatic site = 1
16. number of metastatic site = 2
17. number of metastatic site >=3
18. unknown number of metastatic site
19. treatment start year 2017-2019
20. treatment start year 2020-2022
21. median follow up time, (months)
-0.4
0.0
0.4
Standardized Difference
a.
Unadjusted
After sIPTW
Figure S1. Standardized differences before and after sIPTW in HER2-low and HER2-zero cohorts

## Slide 2
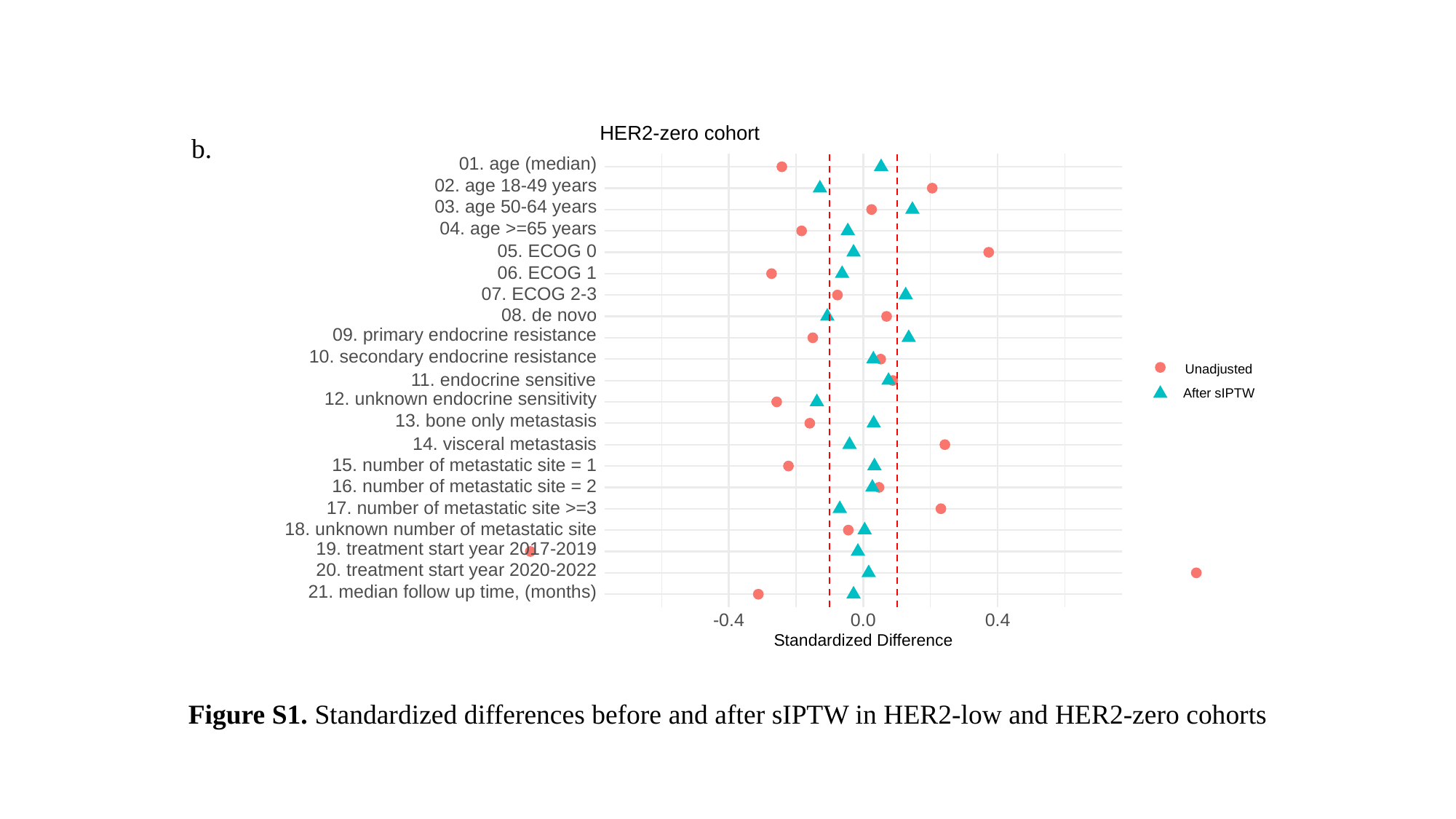

HER2-zero cohort
01. age (median)
02. age 18-49 years
03. age 50-64 years
04. age >=65 years
05. ECOG 0
06. ECOG 1
07. ECOG 2-3
08. de novo
09. primary endocrine resistance
10. secondary endocrine resistance
11. endocrine sensitive
12. unknown endocrine sensitivity
13. bone only metastasis
14. visceral metastasis
15. number of metastatic site = 1
16. number of metastatic site = 2
17. number of metastatic site >=3
18. unknown number of metastatic site
19. treatment start year 2017-2019
20. treatment start year 2020-2022
21. median follow up time, (months)
-0.4
0.0
0.4
Standardized Difference
b.
Unadjusted
After sIPTW
Figure S1. Standardized differences before and after sIPTW in HER2-low and HER2-zero cohorts
