# Supplementary material for: Clinical Outcomes of HER2-Low Versus HER2-Zero in HR-Positive Metastatic Breast Cancer Treated With Endocrine Therapy With or Without CDK4/6 Inhibitors: A Multicenter Retrospective Study
Source: Int J Breast Cancer. 2025 Nov 4;2025:5597051. doi: 10.1155/ijbc/5597051 (PMC12605866; doi:10.1155/ijbc/5597051)
Supplement: Supporting Information 2 — Figure S2: Differences in Kaplan–Meier curves for progression-free survival and overall survival between the HER2-low and HER2-zero cohorts in patients receiving CDK4/6 + AI and AI alone. [file 5597051.f2.pptx]

## Slide 1
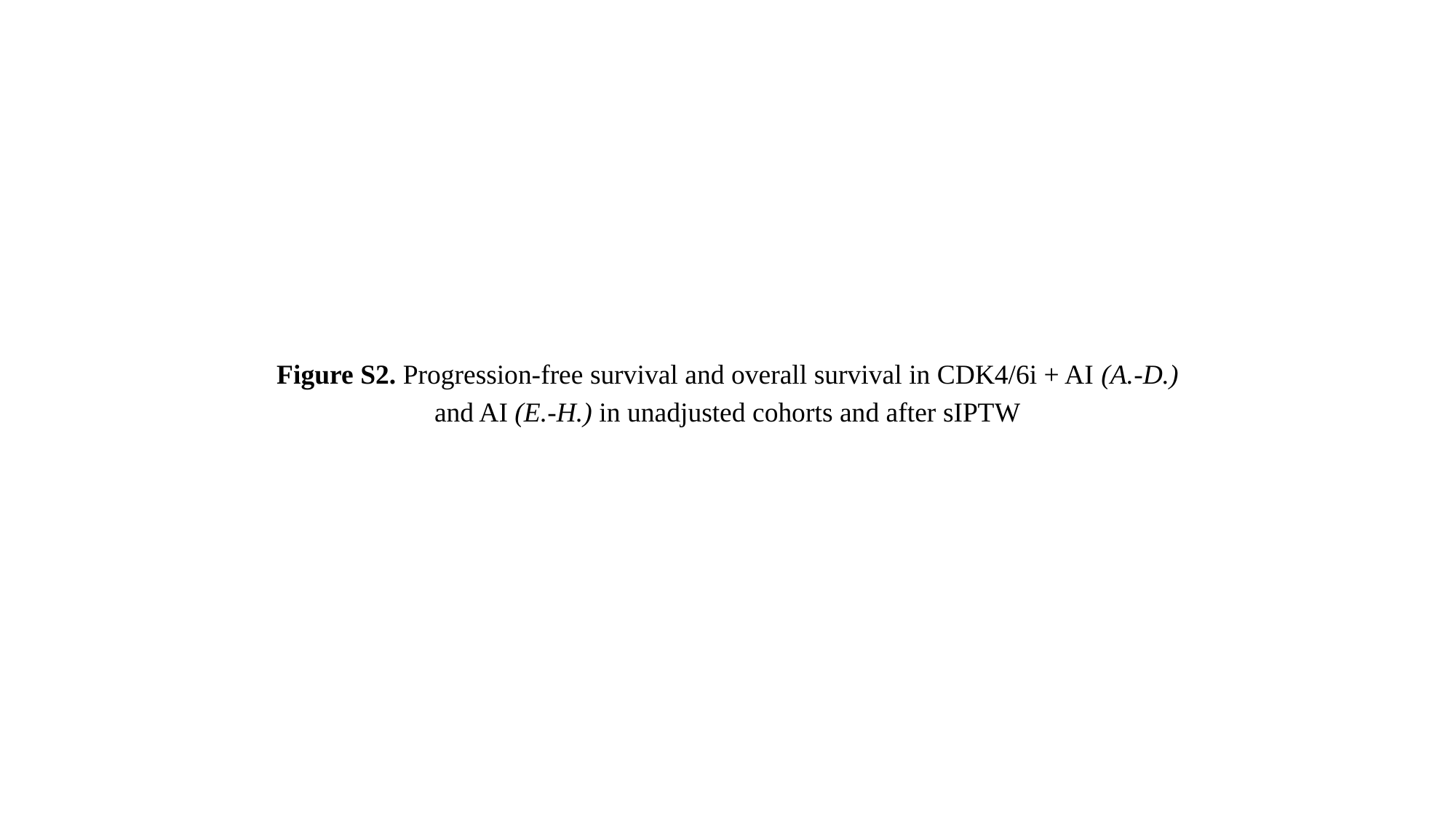

Figure S2. Progression-free survival and overall survival in CDK4/6i + AI (A.-D.) and AI (E.-H.) in unadjusted cohorts and after sIPTW

## Slide 2
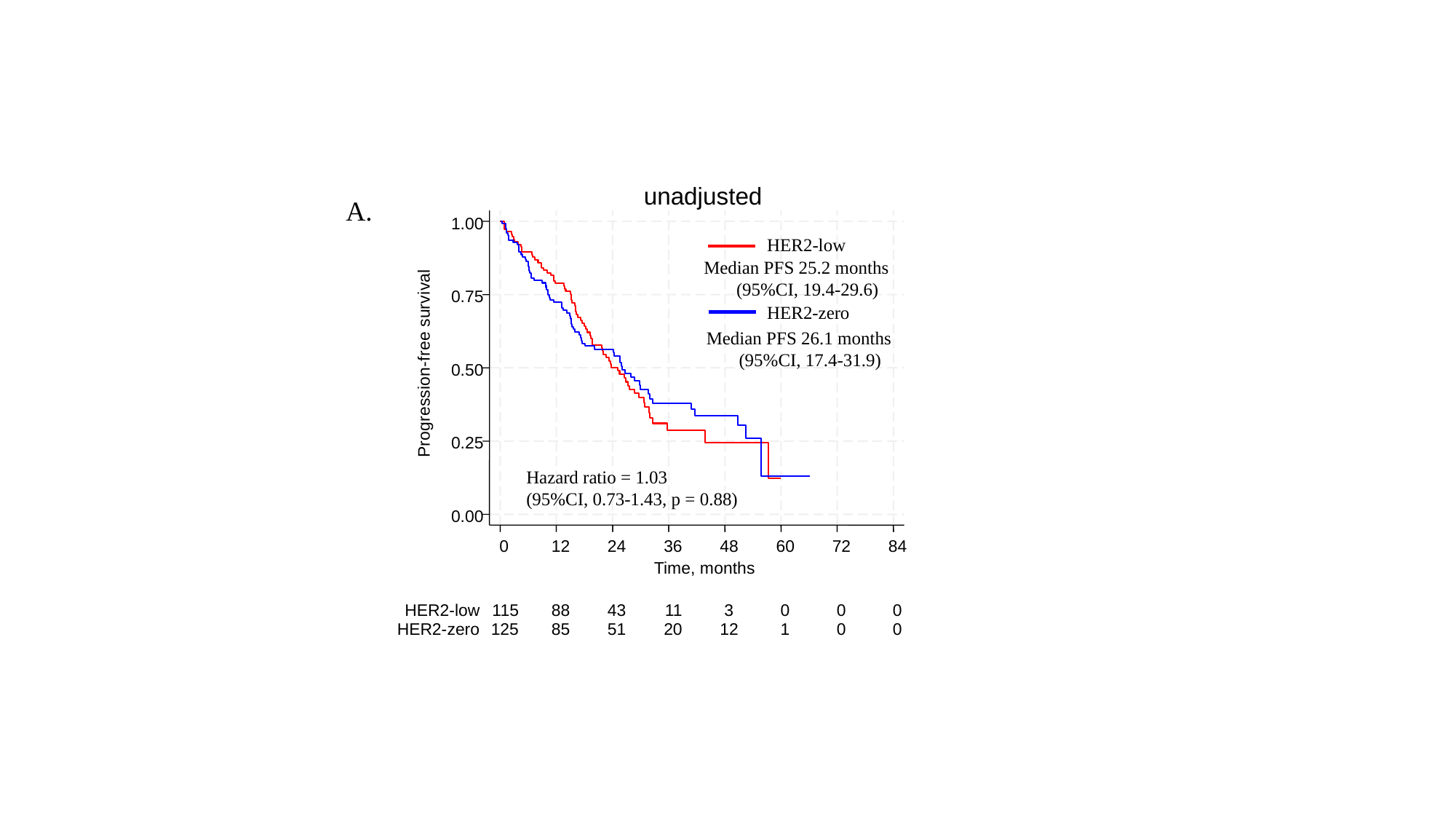

unadjusted
1.00
l
a
v
i
0.75
v
r
u
s
e
e
r
f
-
0.50
n
o
i
s
s
e
r
g
o
r
0.25
P
0.00
0
12
24
36
48
60
72
84
HER2-low
115
88
43
11
3
0
0
0
HER2-zero
125
85
51
20
12
1
0
0
Time, months
A.
HER2-low
Median PFS 25.2 months
(95%CI, 19.4-29.6)
HER2-zero
Median PFS 26.1 months
(95%CI, 17.4-31.9)
Hazard ratio = 1.03
(95%CI, 0.73-1.43, p = 0.88)

## Slide 3
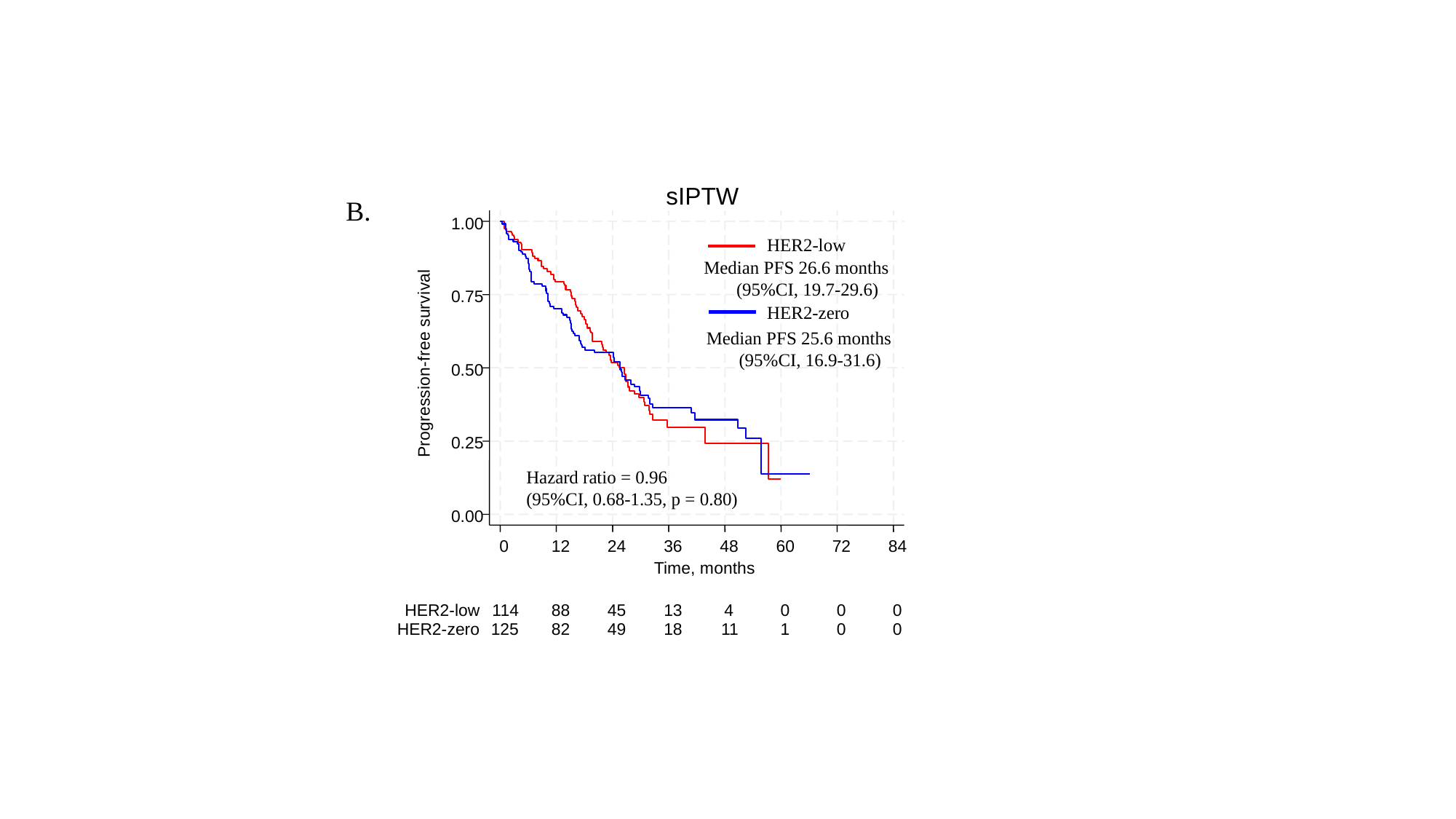

1.00
l
a
v
i
0.75
v
r
u
s
e
e
r
f
-
0.50
n
o
i
s
s
e
r
g
o
r
0.25
P
0.00
0
12
24
36
48
60
72
84
114
88
45
13
4
0
0
0
125
82
49
18
11
1
0
0
Time, months
sIPTW
B.
HER2-low
Median PFS 26.6 months
(95%CI, 19.7-29.6)
HER2-zero
Median PFS 25.6 months
(95%CI, 16.9-31.6)
Hazard ratio = 0.96
(95%CI, 0.68-1.35, p = 0.80)
HER2-low
HER2-zero

## Slide 4
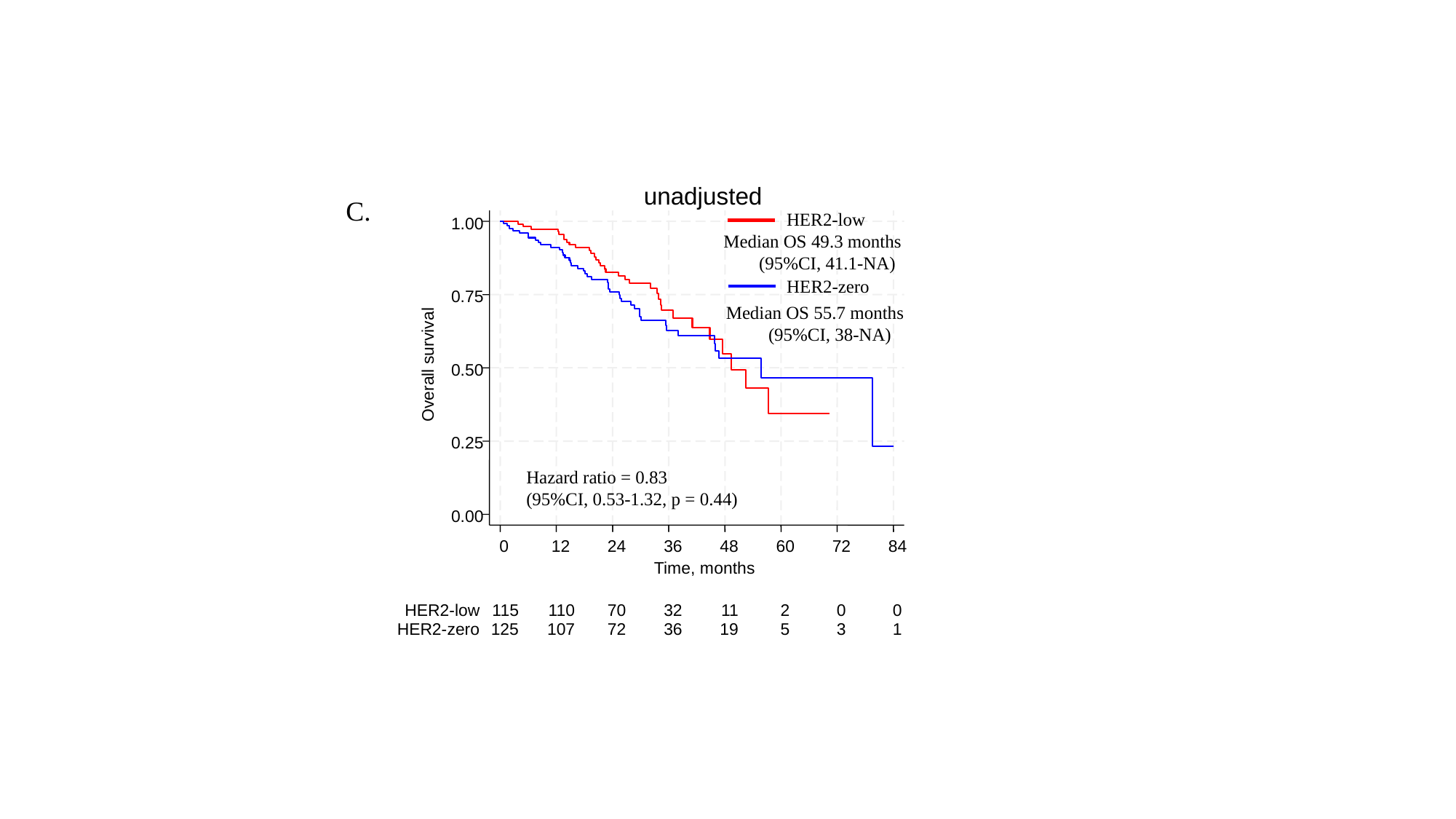

unadjusted
1.00
0.75
0.50
0.25
0.00
0
12
24
36
48
60
72
84
Time, months
115
110
70
32
11
2
0
0
125
107
72
36
19
5
3
1
C.
HER2-low
Median OS 49.3 months
(95%CI, 41.1-NA)
HER2-zero
Median OS 55.7 months
(95%CI, 38-NA)
Overall survival
Hazard ratio = 0.83
(95%CI, 0.53-1.32, p = 0.44)
HER2-low
HER2-zero

## Slide 5
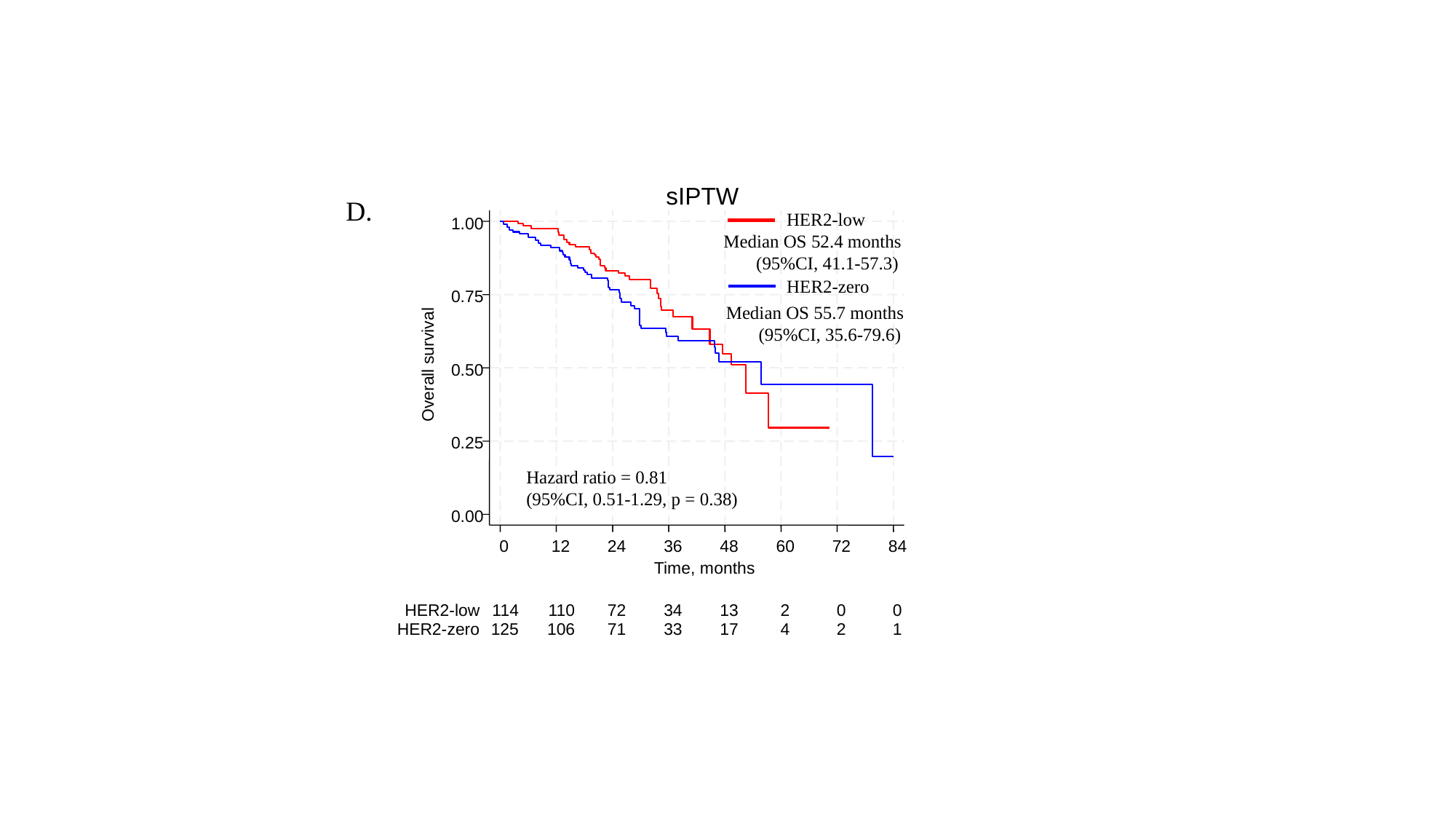

1.00
0.75
0.50
0.25
0.00
0
12
24
36
48
60
72
84
Time, months
114
110
72
34
13
2
0
0
125
106
71
33
17
4
2
1
sIPTW
D.
HER2-low
Median OS 52.4 months
(95%CI, 41.1-57.3)
HER2-zero
Median OS 55.7 months
(95%CI, 35.6-79.6)
Overall survival
Hazard ratio = 0.81
(95%CI, 0.51-1.29, p = 0.38)
HER2-low
HER2-zero

## Slide 6
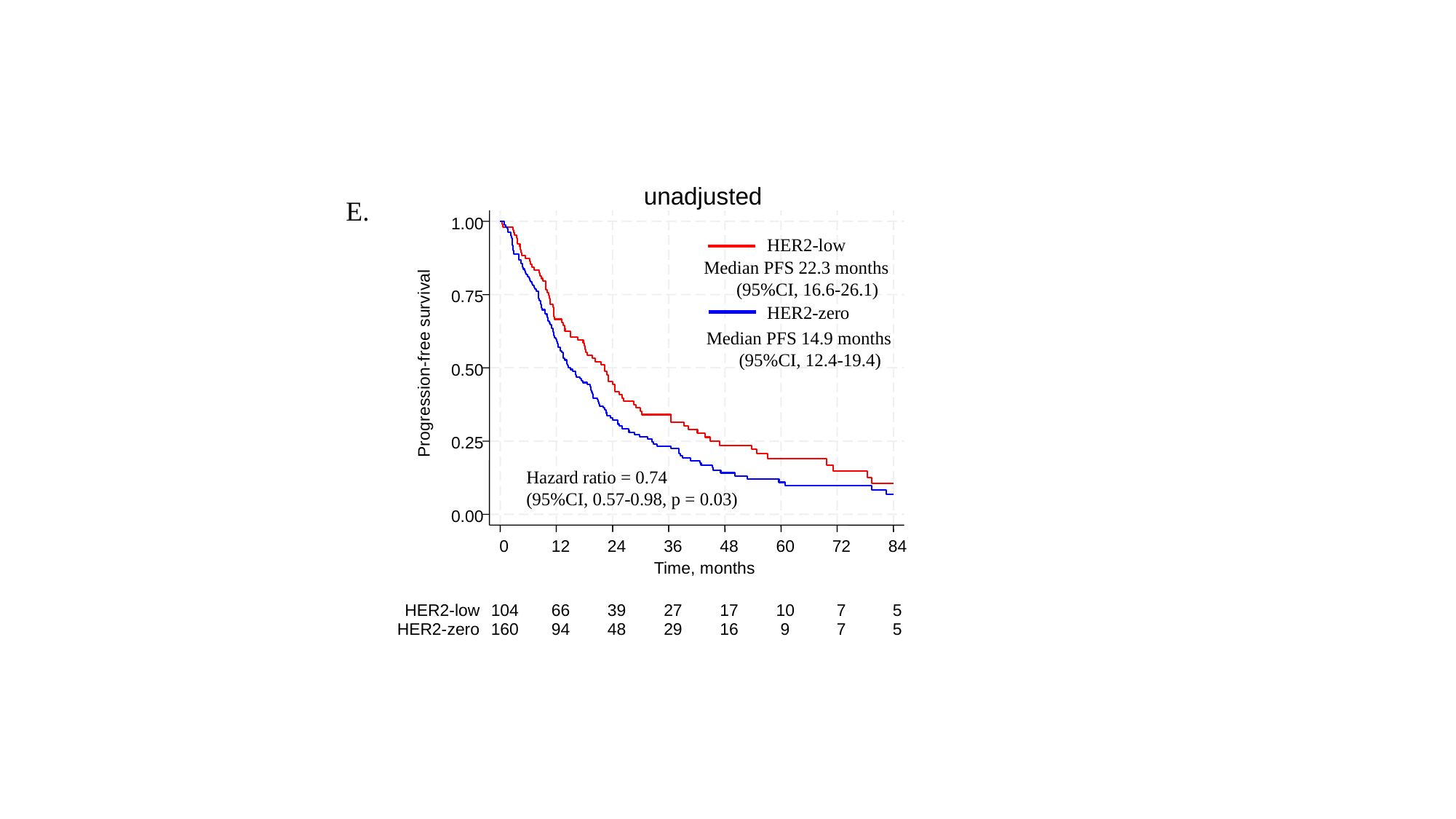

unadjusted
1.00
l
a
v
i
0.75
v
r
u
s
e
e
r
f
-
0.50
n
o
i
s
s
e
r
g
o
r
0.25
P
0.00
0
12
24
36
48
60
72
84
104
66
39
27
17
10
7
5
160
94
48
29
16
9
7
5
Time, months
E.
HER2-low
Median PFS 22.3 months
(95%CI, 16.6-26.1)
HER2-zero
Median PFS 14.9 months
(95%CI, 12.4-19.4)
Hazard ratio = 0.74
(95%CI, 0.57-0.98, p = 0.03)
HER2-low
HER2-zero

## Slide 7
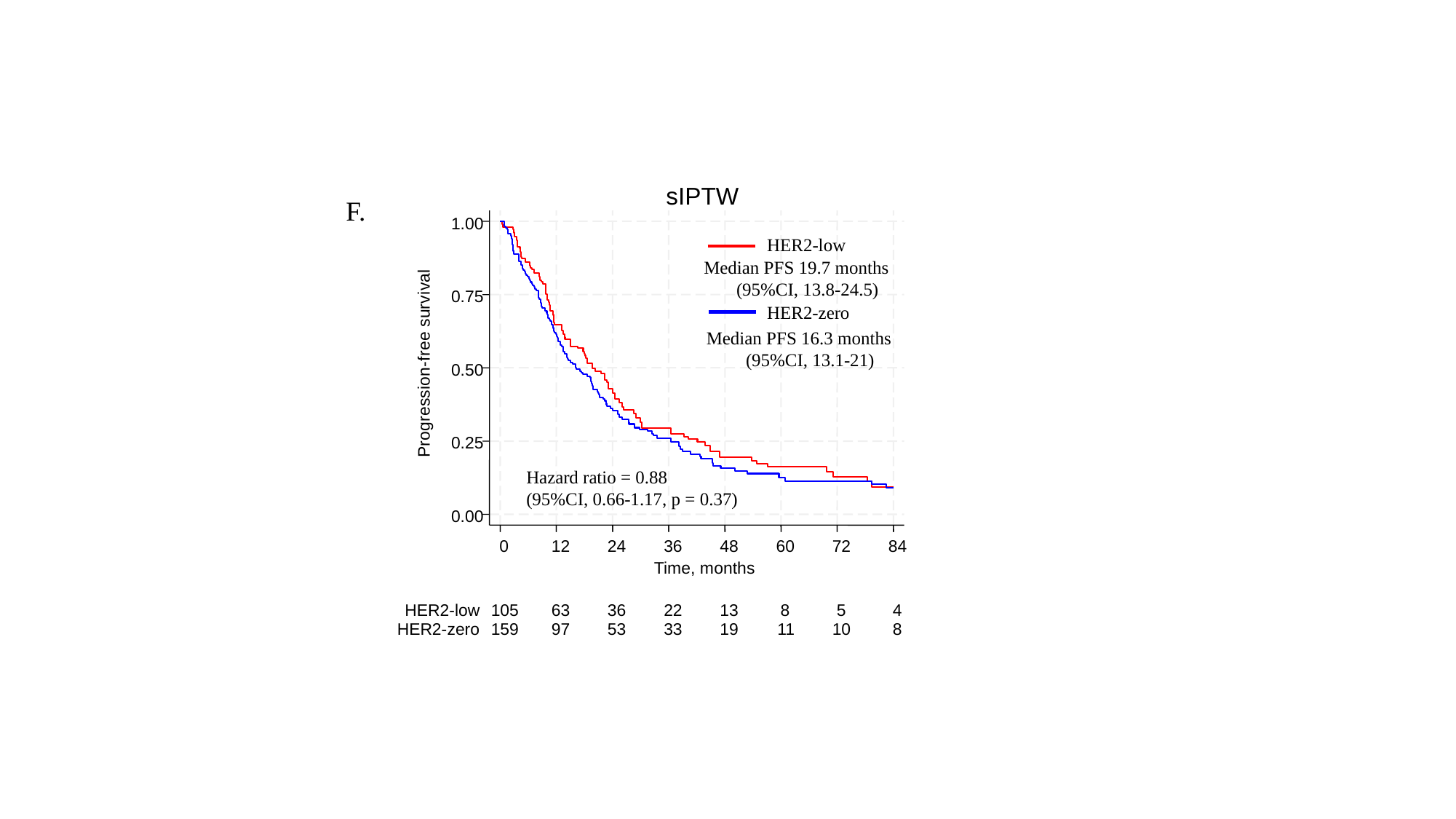

1.00
l
a
v
i
0.75
v
r
u
s
e
e
r
f
-
0.50
n
o
i
s
s
e
r
g
o
r
0.25
P
0.00
0
12
24
36
48
60
72
84
105
63
36
22
13
8
5
4
159
97
53
33
19
11
10
8
Time, months
sIPTW
F.
HER2-low
Median PFS 19.7 months
(95%CI, 13.8-24.5)
HER2-zero
Median PFS 16.3 months
(95%CI, 13.1-21)
Hazard ratio = 0.88
(95%CI, 0.66-1.17, p = 0.37)
HER2-low
HER2-zero

## Slide 8
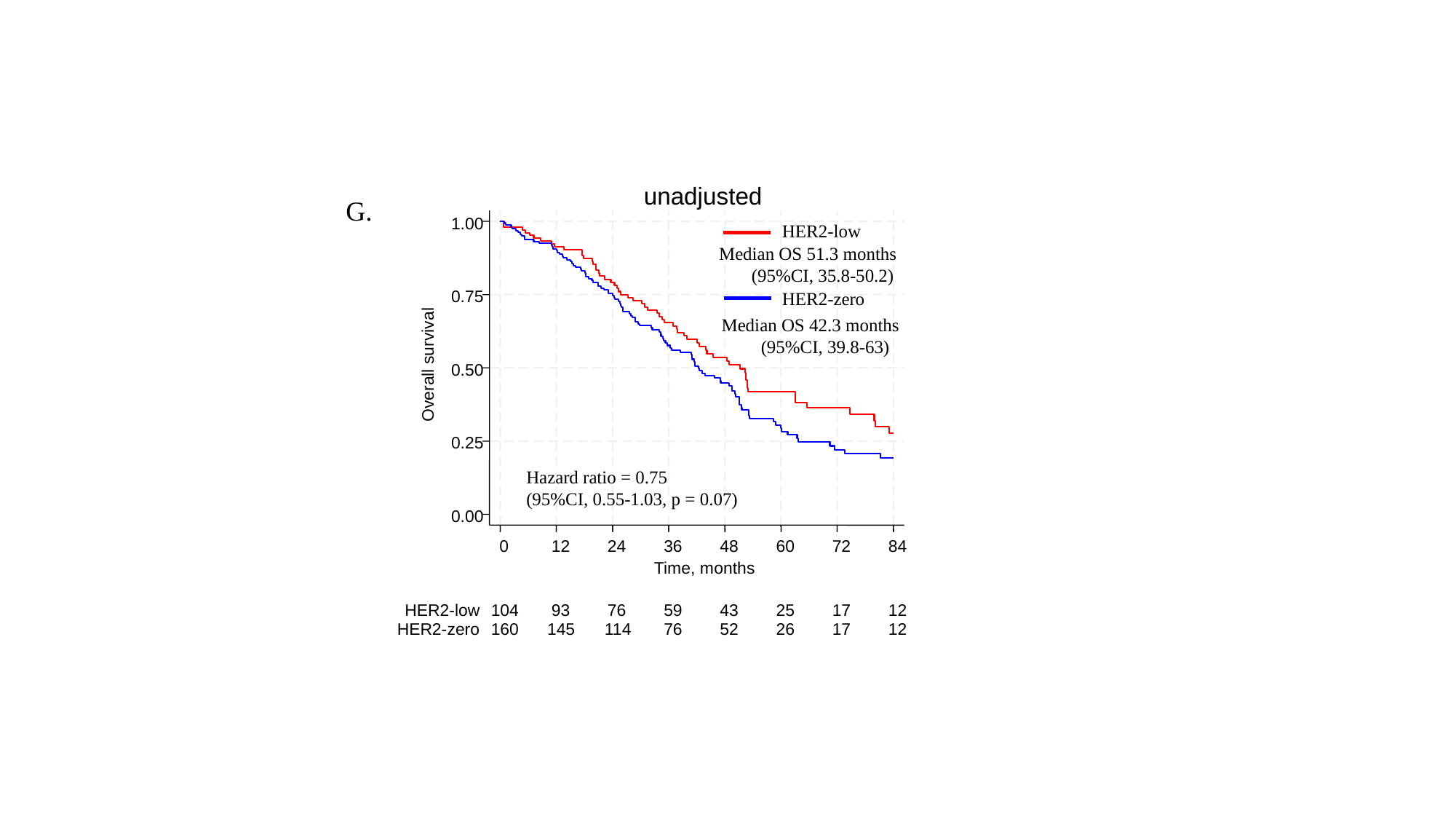

unadjusted
1.00
0.75
0.50
0.25
0.00
0
12
24
36
48
60
72
84
Time, months
104
93
76
59
43
25
17
12
160
145
114
76
52
26
17
12
G.
HER2-low
Median OS 51.3 months
(95%CI, 35.8-50.2)
HER2-zero
Median OS 42.3 months
(95%CI, 39.8-63)
Overall survival
Hazard ratio = 0.75
(95%CI, 0.55-1.03, p = 0.07)
HER2-low
HER2-zero

## Slide 9
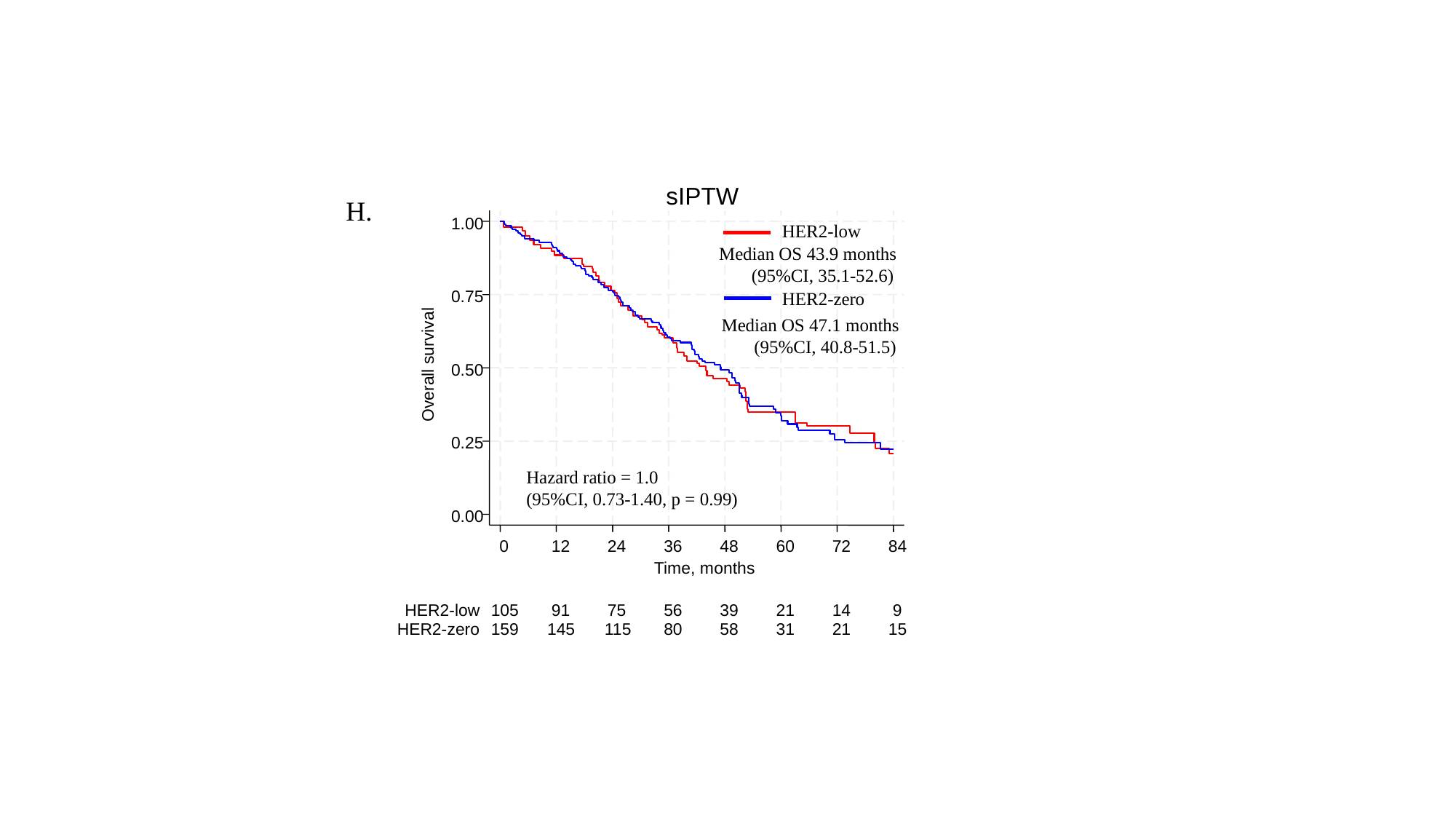

1.00
0.75
0.50
0.25
0.00
0
12
24
36
48
60
72
84
Time, months
105
91
75
56
39
21
14
9
159
145
115
80
58
31
21
15
sIPTW
H.
HER2-low
Median OS 43.9 months
(95%CI, 35.1-52.6)
HER2-zero
Median OS 47.1 months
(95%CI, 40.8-51.5)
Overall survival
Hazard ratio = 1.0
(95%CI, 0.73-1.40, p = 0.99)
HER2-low
HER2-zero
