# Supplementary material for: Clinical Outcomes of HER2-Low Versus HER2-Zero in HR-Positive Metastatic Breast Cancer Treated With Endocrine Therapy With or Without CDK4/6 Inhibitors: A Multicenter Retrospective Study
Source: Int J Breast Cancer. 2025 Nov 4;2025:5597051. doi: 10.1155/ijbc/5597051 (PMC12605866; doi:10.1155/ijbc/5597051)
Supplement: Supporting Information 3 — Figure S3: The standardized difference values before and after sIPTW in the CDK4/6i + AI and AI alone cohorts. [file 5597051.f3.pptx]

## Slide 1
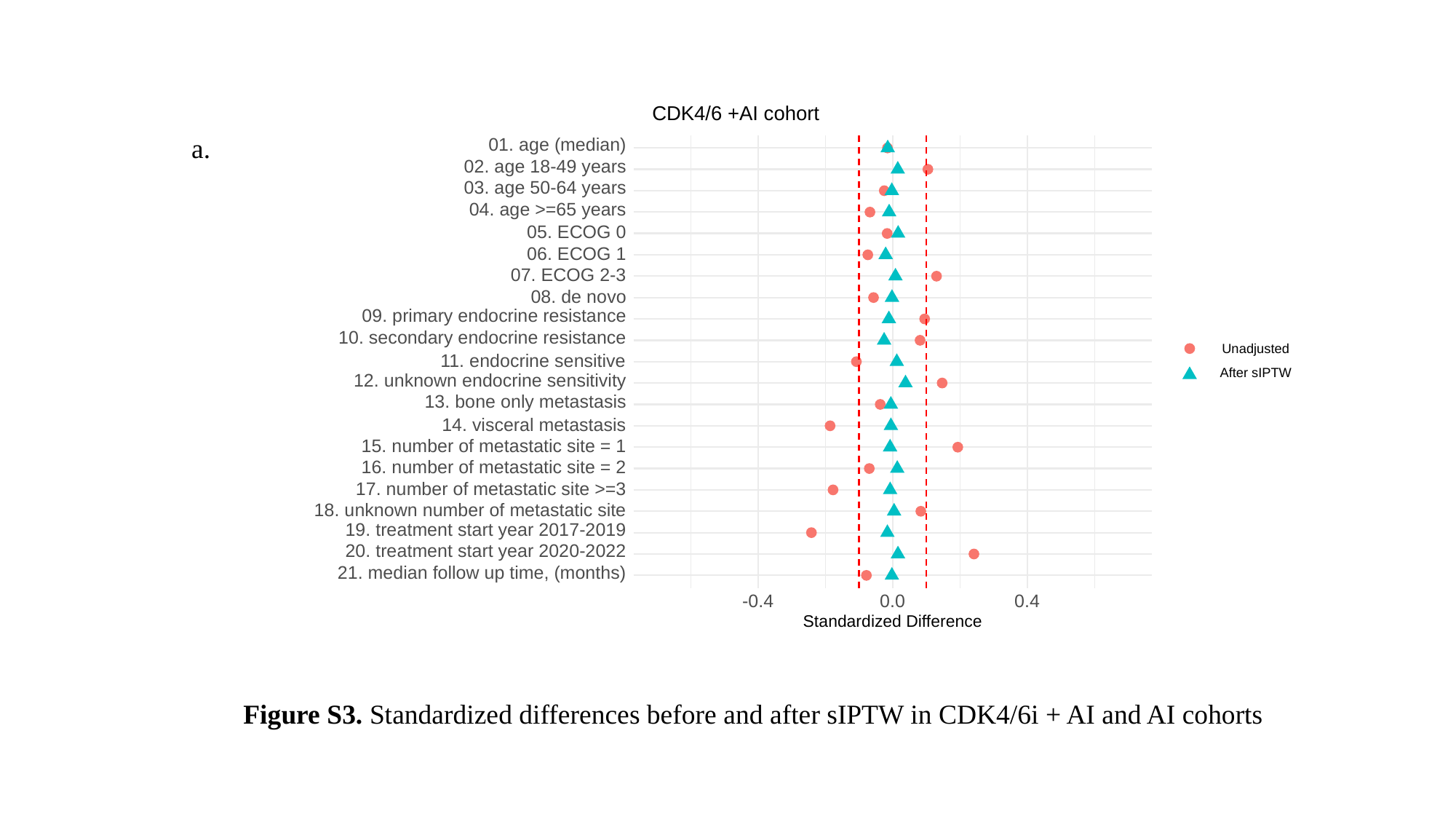

CDK4/6 +AI cohort
01. age (median)
02. age 18-49 years
03. age 50-64 years
04. age >=65 years
05. ECOG 0
06. ECOG 1
07. ECOG 2-3
08. de novo
09. primary endocrine resistance
10. secondary endocrine resistance
Unadjusted
11. endocrine sensitive
After sIPTW
12. unknown endocrine sensitivity
13. bone only metastasis
14. visceral metastasis
15. number of metastatic site = 1
16. number of metastatic site = 2
17. number of metastatic site >=3
18. unknown number of metastatic site
19. treatment start year 2017-2019
20. treatment start year 2020-2022
21. median follow up time, (months)
-0.4
0.0
0.4
Standardized Difference
a.
Figure S3. Standardized differences before and after sIPTW in CDK4/6i + AI and AI cohorts

## Slide 2
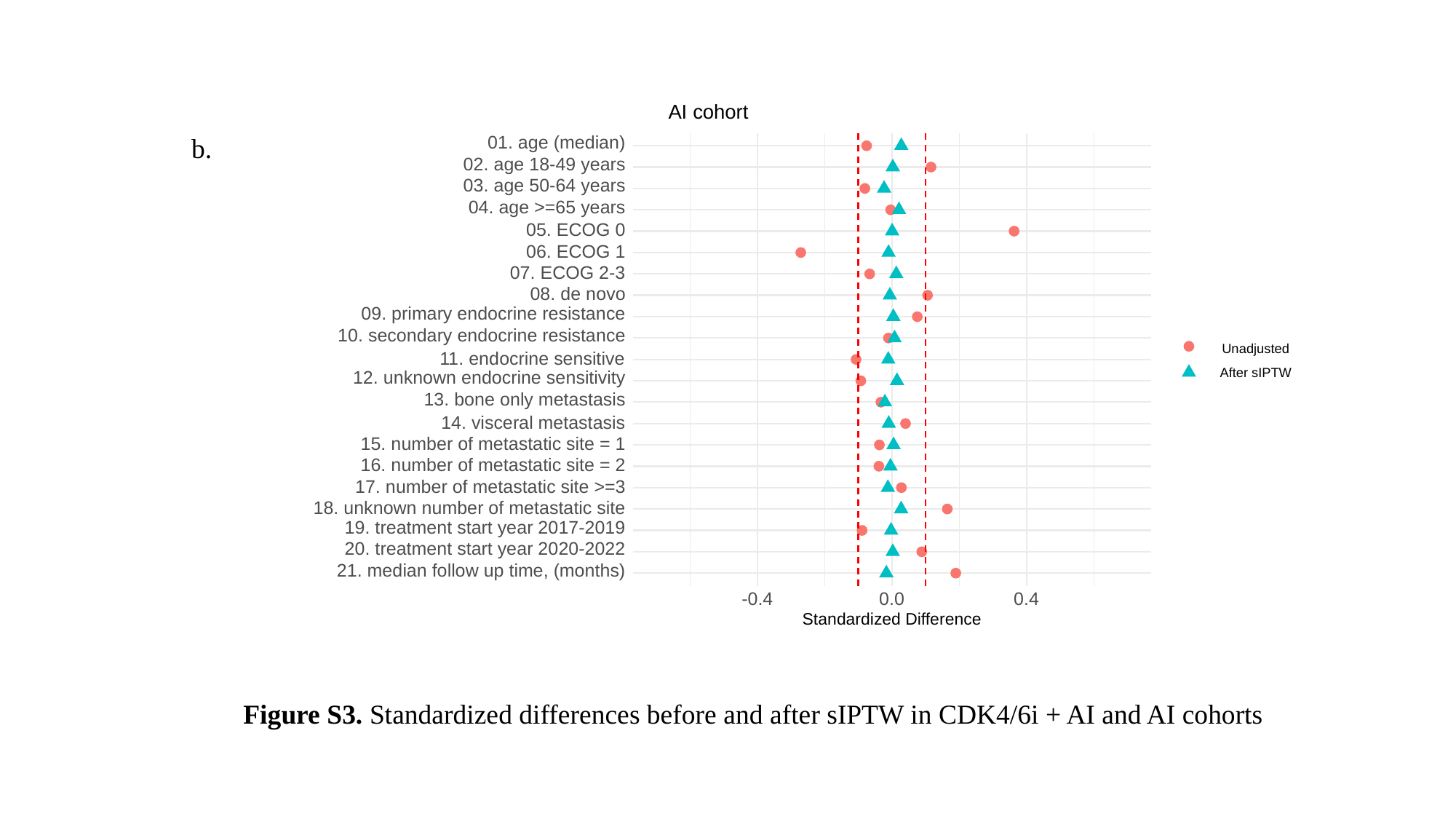

AI cohort
01. age (median)
02. age 18-49 years
03. age 50-64 years
04. age >=65 years
05. ECOG 0
06. ECOG 1
07. ECOG 2-3
08. de novo
09. primary endocrine resistance
10. secondary endocrine resistance
11. endocrine sensitive
12. unknown endocrine sensitivity
13. bone only metastasis
14. visceral metastasis
15. number of metastatic site = 1
16. number of metastatic site = 2
17. number of metastatic site >=3
18. unknown number of metastatic site
19. treatment start year 2017-2019
20. treatment start year 2020-2022
21. median follow up time, (months)
-0.4
0.0
0.4
Standardized Difference
b.
Unadjusted
After sIPTW
Figure S3. Standardized differences before and after sIPTW in CDK4/6i + AI and AI cohorts
